# A Qualitative Inquiry to Understand the Impact of a Facility Dog on Hospital Workers’ Perceived Organizational Support

**DOI:** 10.1177/00469580261417528

**Published:** 2026-01-29

**Authors:** Jaskiran Baweja, Jason B. Coe, Maziha Kamal, Janine Noorloos, Marianne Saragosa, Behdin Nowrouzi-Kia, Basem Gohar

**Affiliations:** 1 University of Guelph, ON, Canada; 2 Sinai Health, Toronto, ON, Canada; 3 University of Toronto, ON, Canada; 4 Laurentian University, Sudbury, ON, Canada

**Keywords:** organizational support, facility dog, hospital staff, healthcare, employee support

## Abstract

Health systems are under pressure due to staffing shortages and retention challenges, exacerbated by COVID-19. Its negative impact on hospital staff’s well-being, coupled with limited support, highlights the need for more innovative organizational solutions to improve retention. Recognizing this, an Ontario hospital became the first in Canada to employ a full-time facility dog to support hospital staff in distress. This qualitative study aimed to explore the experiences of hospital staff with a facility dog and its impact on the staff’s perceived organizational support. One-on-one, semi-structured interviews were conducted with clinical, non-clinical and volunteer staff at the hospital. Data were audio-recorded, transcribed, de-identified, and then thematically analyzed. The sample comprised 27 participants, including 13 clinical and 12 non-clinical employees, and 2 volunteers, with the majority identifying as women (77.78%). The results revealed the following themes: (1) the facility dog offers a unique type of support that is distinct and innovative from currently available support; (2) the facility dog’s presence has made positive organizational changes; (3) the facility dog provides a short-term relief that may be limited to some staff roles; (4) a sense of pride in being leaders by introducing such program to a Canadian hospital; (5) the need for organizational improvements beyond the facility dog. Participants perceived the facility dog as having a positive, although short-term, impact, particularly among non-clinical employees. The results suggest that while the dog can improve perceived organizational support, additional long-term initiatives are needed to maintain these perceptions and support over time.

## Introduction

The current healthcare system is under pressure due to rising staffing shortages and retention challenges, which the COVID-19 pandemic has exacerbated.^1^ Staffing shortages have contributed to an increased workload for healthcare staff, negatively affecting their physical and mental well-being, and consequently leading to an increase in sickness absenteeism and intentions to leave the profession.^2,3^ For example, a study by Nakweenda et al^4^ found that heavy workloads and inconsistent breaks left hospital employees fatigued, stressed, and dissatisfied, resulting in increased sick leave. Unsurprisingly, the absence of perceived support in the workplace has been found to increase the odds of perceived poor mental health.^5^

While factors such as heavy workloads can be difficult to address, other organizational factors can enhance wellbeing, reduce sick leave, and increase retention. For instance, results from a meta-analysis revealed that perceived organizational support can lower the odds of sickness absence among nursing personnel.^2^ Similarly, Pattali et al^6^ revealed that transformational leadership reduced the odds of intentions to leave the profession, moderated by perceived organizational support.

Among the novel methods of support, based on existing literature, that can be used to improve workplace wellbeing, is the inclusion of animals. The human-animal relationship is well-studied.^7^ Although this field lacks a universal theoretical foundation,^8^ evidence shows that humans and domesticated animals derive mutual psychological benefits. Growing research also supports the therapeutic value of canines, particularly for patients and, to some extent, employees. For instance, 1 study found that first responders experienced immediate stress relief, comfort, happiness, calm, and positive distraction from work through interactions with canine support.^9^ Another study reported nearly 50% improvement in mood scores among clinical staff after interacting with a therapy dog, as well as a significant decrease in client-related burnout.^10^ Similarly, several studies in pediatric hospitals show that facility dogs, working alongside their handlers, provide psychosocial support to patients and families and offer well-being benefits for hospital staff, including improved mood, morale, and reduced intentions to quit.^11-14^

Recognizing the potential benefits of human-animal interactions, particularly with dogs and with the aim to improve their organizational climate, Cambridge Memorial Hospital (CMH) in Ontario, Canada, became the first hospital nationally to employ a full-time National Service Dog (NSD) certified facility dog, Ember, who was brought in to support hospital staff in distress. The dog was trained with specific commands given by handlers, including, but not limited to, resting her chin on a staff member’s lap and hugging the staff. Facility dogs are essentially certified dogs trained with specific skills for a particular facility.^15^

The facility dog arrived at CMH in May 2022 and began working in August 2022, providing support exclusively to CMH staff and volunteers.^16^ Her work routine consists of making daily rounds in various hospital units, accompanied by 1 of her 4 handlers, who are trained and certified by NSD. She works regular full-time hours, Monday through Friday and is “on-call” when needed, which staff can request by contacting her handler. When not working at CMH, she spends her time living with her primary handler.

While human-animal interactions and pet therapy can enhance well-being, research on their organizational impact and on facility dogs in Canadian healthcare remains limited. The purpose of this study was to explore the perceptions of hospital staff toward Ember and her impact on their perceived level of support.

## Methods

Approval from the University of Guelph’s Ethics Research Board was obtained (REB#23-10-003). The findings of this qualitative study were reported using the Consolidated Criteria for Reporting Qualitative Research (COREQ)^17^ checklist (Supplemental Appendix 1).

### Participants and Setting

This qualitative descriptive study was conducted among CMH employees using a purposive sampling approach.^18^ The leadership team at CMH distributed the study invitations to employees on behalf of the research team through posted ads and emails. Interested participants contacted the research team and were recruited directly, provided they met the study’s eligibility criteria, which included being 18 years or older, English-speaking, and employees or volunteers working full-time or part-time at CMH in a clinical or non-clinical capacity. The senior researcher (BG), introduced the study and answered questions from participants before the study began. “Non-clinical” refers to employees who do not provide direct patient care (eg, maintenance, human resources). To gain a broader perspective on the NSD program, participants were eligible regardless of their level of interaction with the facility dog since joining CMH 2 years prior. This inclusive approach enabled participants to share their reasons for engaging with or not engaging with a facility dog. Individuals under 18 years of age or those who were no longer employed at CMH during the study period were ineligible. Informed consent was obtained in writing from all eligible participants before participating, and they were scheduled based on mutual availability.

### Procedures

Data were collected through semi-structured, one-on-one interviews conducted by the senior researcher (BG), who has extensive experience in qualitative research, accompanied by a research assistant (AW). The interviews were conducted virtually from May to September 2024 in English via Microsoft Teams (MT), until data saturation was achieved, considering both clinical and non-clinical roles. All participants received a $50 (CAD) Amazon e-gift card as an incentive.

The participants were asked to provide general demographic information and their job descriptions. Semi-structured interviews explored (1) their experiences working at CMH, (2) their experiences with Ember, (3) their perceptions of the CMH leading this initiative, (4) Ember’s impact on their perceived organizational support, and (5) suggestions for future organizational opportunities and improvements (Supplemental Appendix 2). The interviews were audio-recorded, then transcribed verbatim using MT. Next, JB reviewed the transcripts’ accuracy by cross-checking transcripts with the audio data, and then the transcripts were de-identified for analysis.

### Data Analysis

Data were analyzed using the broad structure of thematic analysis^19^; however, we applied a codebook approach, which involved open coding of the data to generate codes and identify major themes.^20^ Research team members (JB, MK, JN) applied an inductive-deductive approach for primary coding, where they independently reviewed and coded 8 transcripts. The common codes from the 8 transcripts were then discussed and compiled into a codebook, which was later used deductively by the research team to analyze the remaining transcripts using the software Dedoose.^21^ To maintain rigor throughout the process, the research team reviewed each other’s codes as a secondary check to ensure consistent coding throughout the analysis. The codebook was iteratively updated to incorporate new codes as they emerged from the data. The research team conducted a final review of the codes, where they discussed potential discrepancies and classified major themes from the data. They also maintained an audit trail with detailed records of the data collected and analyzed, ensuring the dependability of the findings.

## Results

Data saturation was achieved after 27 interviews (N = 27). To help preserve the participants’ identities, descriptive information is provided more broadly, within ranges (Table 1). The sample consisted of 13 clinical and 14 non-clinical participants, including 2 volunteers, with most identifying as women. Most participants fell within the 20 to 35 age group. The average interview length was 27:27 min (range: 16:00-42:16).

**Table 1.** Participant Characterstics.

Characteristics	Participants n (%)
Age range (years)	20-35 = 15 (55.6%)
	36-51 = 9 (33.3%)
	51+ = 3 (11.1%)
Job role	Clinical = 13 (48.2%);
	Non-clinical = 12 (44.4%);
	Volunteers = 2 (7.4%)
Gender	Woman = 21 (77.8%);
	Man = 6 (22.2%)

Our results revealed 5 key themes: (1) the facility dog offers a unique type of support that is distinct and innovative from currently available support; (2) the facility dog’s presence has made positive organizational changes; (3) the facility dog provides a short-term relief that may be limited to some staff roles; (4) a sense of pride in being leaders by introducing such program to a Canadian hospital; (5) the need for organizational improvements beyond the facility dog (Table 2).

**Table 2.** Major Themes and Sub-Themes Drawn from the Data.

Major themes	Sub-themes
Theme 1: The facility dog offers a unique type of support that is distinct and innovative from currently available support	Sub-theme 1: Immediate support that helps you stay “in the moment”
	Sub-theme 2: A multisensory support, empathetic attunement and emotional response without saying a word
Theme 2: The facility dog’s presence has made positive organizational changes	Sub-theme 1: Community Building
	Sub-theme 2: Normalizing microbreaks
Theme 3: The facility dog provides short-term relief that may be limited to some staff roles	Sub-theme 1: The facility dog provides a short-term relief from work-related stress
	Sub-theme 2: Barriers in accessing the facility dog based on job roles
Theme 4: A sense of pride in being leaders by introducing the NSD program to a Canadian hospital	Sub-theme 1: Staff feel pride in CMH offering an innovative initiative
	Sub-theme 2: Staff feel valued by CMH
Theme 5: The need for organizational improvements beyond the facility dog	

## Theme 1: The Facility Dog Offers a Unique Type of Support That is Distinct and Innovative from Currently Available Support

Ember’s support was described as a non-traditional type of support, an uncommon experience for the participating CMH employees. Participants expressed that Ember offered immediate support through a multisensory approach that was inclusive to employees regardless of their emotional needs.

### Subtheme 1: Immediate Support That Helps You Stay “in the Moment”

Several participants reported that having Ember provided them with unique and non-traditional support at work, making them feel confident in expressing their emotional vulnerability. They felt that Ember’s presence offered immediate support regardless of the time or nature of the challenges they experienced at work.

P19 (clinical) But I know for lots of folks, probably including myself, I might be more hesitant if it's someone that works for the organization, I might be hesitant to share my feelings because you don't want to misspeak about a colleague or you don't want to say something that maybe would put you in a compromised position. Whereas with Ember, you don't have to worry the same way about the intentions of or about what the kind of prolonged outcome could be if you were to voice frustration

P15 (non-clinical) We also have the chance to say, ‘Hey, we want her today at 3:00 o'clock because something might have happened in our department, and we just want her to come there for that.’ So that's nice to have as well. It's not just when it's on her time, it's also on our time.

Participants also noted that, in addition to being able to seek support sooner, they reported the convenience of instant support that helps them refocus “in the moment”

P16 (clinical) Ember came right over and like put her head on me like and it was like a very simple gesture but it was like kind of in that moment that I realized, like oh yeah, I'm actually, am really frustrated and it kind of took this thing happening to realize, like, yeah, I need to take a minute to actually to debrief because I am frustrated with the situation. And I've found that to like, that sticks out in my head as very significant, as it really did make me feel supported in that moment. And it kind of provided something that I don't think a colleague would have been able to provide.

### Subtheme 2: A Multisensory Support, Empathetic Attunement and Emotional Response Without Saying a Word

Participants reported that their communication with Ember was multi-sensory. They felt that hugging Ember, having Ember rest her head on their knee, along with the empathetic look in her eyes, positively impacted their emotional well-being, making them feel supported. They reported experiencing comfort by touching Ember. This form of comfort was particularly described as advantageous following critical incidents where some participants reported experiencing heightened emotions and the need to express their grief and disappointment without fearing judgment, for which Ember offered an outlet. The participants described feeling supported by Ember without having to articulate the challenges they experienced. They described feeling an empathetic connection, as her mere presence, body language, and facial expressions created a space for them to acknowledge their feelings and feel supported, even without speaking. They also described feeling a sense of comfort and familiarity in her peaceful demeanor, which further supported them during challenging debriefs.

P25 (clinical) She's just, I don't know. You look at her. She's so sweet. Like she makes me melt. And it's so weird because like I said, I'm really not a dog person.

P23 (clinical) She has those eyes when she stares at you, when she looks up at you, the eye contact, I think is a big thing!

P7 (clinical) It's sometimes nice just to sit with the dog and not have to explain or talk or do anything, just kind of being with an animal for me is really helpful. . .

## Theme 2: The Facility Dog’s Presence Has Made Positive Organizational Changes

Participants expressed that Ember’s presence was a positive change in the workplace, offering community building and normalizing a culture of microbreaks. Many participants reported that Ember created opportunities for frequent social engagement among hospital staff.

### Subtheme 1: Community Building

Participants reported that Ember’s presence created a sense of community among staff by promoting increased socializing, including engagements with her handlers. They expressed that watching Ember interact with CMH employees and volunteers evoked a sense of belonging for many, as they felt they could easily connect with their colleagues by sharing their experiences related to Ember. Participants described Ember as a valuable member of the organization who increased staff engagement and allowed them to bond through small talk about Ember and their own pets, despite their busy workload.

P22 (clinical) And others just stopping to come over, bending down, you know, chin scratch, head scratch, maybe sitting and letting her interact a little bit. And just some conversation with the trainer of just about things that are going on with their, a few questions about her, and just taking a time out.

P26 (clinical) But if it's in the hallway, I feel like I bring that back with me. Oh, I’ve seen Ember, and you know, you get to chat about it, and then maybe someone else brings up a story about their pet or something.

Furthermore, participants expressed that they were able to better understand their team through Ember, as they observed changes in employees’ expressions and body language, which consequently helped them learn when to offer support to their colleagues. Ember’s strong and positive impacts on the staff were evident through her “branding effect.” Participants described how Ember’s contributions to creating a healthy workplace and her shared connection with the staff were promoted through specialized gifts sold at the hospital. These gifts, which were visible every time they were seen, provided a daily reminder of her presence and involvement with the CMH staff.

P4 (non-clinical) . . .but just seeing how they gravitated towards her, patting her, kind of bent down and just having that feeling there, you know, my take on it was helpful for them even more so than hearing about, you know, hearing [Employee Assistance Program] or we're gonna have [Organization] come on site.

P2 (non-clinical). . .we have Ember socks in the gift shop and an Ember plush toy, and Ember serves as a mascot of sorts and so I think there is, you know, there is benefit to taking a second, pausing from work and interacting with Ember, but there's also some build on that community piece that I was speaking to where that, you know, it's something, it's a shared experience that you have with others in the hospital.

### Subtheme 2: Normalizing Microbreaks

Participants also reported that interactions with Ember allowed them to normalize the idea of microbreaks and checking in with their co-workers. They reported that because of Ember, they were able to take frequent breaks without feeling guilty and felt more comfortable and confident in taking breaks. Participants appreciated having Ember as a constant reminder to prioritize taking breaks. They also reported that interactions with Ember helped them incorporate a mindfulness approach into their daily schedule without feeling guilty, promoting a healthy work mindset.

P7 (clinical) . . . Sometimes we're really doing a lot of work and not always social with each other, so it's kind of nice that we're able to kind of take a break together.

P19 (non-clinical) Maybe it's just like the person that's with them also like checking in, asking how your day is, or just like the playfulness of having a dog around in a serious environment, like it just kind of offsets-It's a different vibe. Just more playful. And it's not as serious.

P24 (clinical) Honestly, part of it is a break from what's happening. A moment where I can have a mindful moment, basically where I can forget about everything else and just have that moment where it's like, ‘OK, I can focus on this adorable dog in front of me’ and just take that kind of stress-free moment, that little bit of mindfulness that I can force into my day.

## Theme 3: The Facility Dog Provides Short-Term Relief That May be Limited to Some Staff Roles

Ember’s impact, although powerful in various aspects, was primarily described by participants as short-term. Furthermore, specific job roles and work demands were identified as primary barriers to accessing Ember for some participants, which they highlighted as being the complex nature of her role.

### Subtheme 1: The Facility Dog Provides a Short-Term Relief from Work-Related Stress

Despite the much-needed diversion participants described that Ember offered, they reported that the relaxing benefits are generally temporary, with some describing her impact as a “band-aid effect” or a “good distraction.”

P16 (clinical)Oh, she's gone, and then I'm back to you know, how I felt before seeing her. I'd say like maybe half an hour and then we'll, actually, not even that. Like, I'm estimating that, but it depends on, you know, when I get back to work, so if, let's say another patient comes in and then I just have to focus on helping them out, I might forget about that interaction and just focus on my job.

P27 (clinical)I'd be happy for like a few minutes afterwards until I'm usually back at my job and focusing on the task at hand. But I'll probably have those feelings up until I have to go back to like my job, per se.

### Subtheme 2: Barriers in Accessing the Facility Dog Based on Job Roles

Disparities emerged between clinical and non-clinical participants in engagement with Ember. Clinical staff reported limited interaction due to demanding workloads, scheduling conflicts, staffing shortages, and concerns about infection control, whereas non-clinical staff experienced more frequent visits and interactions.

P24 (clinical) It's one of those things, unfortunately, just because I'm constantly on the go, I feel like I'm constantly with patients. I don't get as much time with Ember as I would have liked.

P6 (clinical) I am happy to see her and everything, but how often I'm finding I'm kind of busy at that time, and so I am actively doing something,so I don't have the time to stop, necessarily, and really spend time with her. But I do think it is a really good thing.

## Theme 4: A Sense of Pride in Being Leaders by Introducing the NSD Program to a Canadian Hospital

Ember’s presence ignited a sense of pride among participants and made them feel valued by their organization. Participants described CMH as having a new identity as pioneers, which they expressed pride in being a part of such a novel initiative.

### Subtheme 1: Staff Feel Pride in CMH Offering an Innovative Initiative

Participants reported feeling proud of the CMH’s innovative approach to supporting staff. They described a feeling of being prioritized by CMH due to its willingness to invest funds and resources in a new, “never done before” initiative to support staff well-being. Participants felt that the leaders of CMH are willing to try new initiatives to support employee well-being. Participants also felt a sense of joy in being part of an organization that sets a high standard for organizational support in the industry. For many participants with previous work experience in other health care organizations, CMH felt like a “breath of fresh air” as they offered support to staff through an “innovative” initiative via Ember.

P16 (clinical) I do think it's very innovative and I think that when Ember first came I was kind of surprised that it was the first like therapy dog for staff. However, I do think that it is a reflection of a lot of the things that CMH does like I think Ember is one piece of the puzzle of the things that CMH does to try to ensure that their staff feel supported.

P10 (clinical) When I think about Cambridge Memorial Hospital choosing to put funding toward something that's going to like help us in the long run, I think that really makes me confident in them as an employer.

P27 (clinical) I'm really happy and proud that we can be an example for other hospitals to show that like instead of like that it can set an example that and hopefully this initiative will be passed on to other hospitals because I do think it is beneficial.

### Subtheme 2: Staff Feel Valued by CMH

Participants reported feeling valued by the organization. They felt that the organization actively worked to understand their needs and offered solutions accordingly. For many participants with previous experience in health care, CMH created an inclusive workplace that valued staff input in decision-making, as CMH brought Ember into the hospital after a staff member suggested employing a facility dog, making staff feel heard. Participants also felt that CMH understood the long-term adverse effects of the high workload that the staff had experienced, especially staff working overnight shifts. So, they ensured that the support offered through Ember was available upon request. This made some participants feel looked after, even when they did not interact with the CMH leaders as often. It highlighted that CMH considered every possible challenge that the staff may experience when offering initiatives.

P10 (clinical) I think it's a way of them showing that they realize there are some gaps in like the stressfulness of working in healthcare, but also them showing they have foresight to want to help us, and I think it is helping in the end.”P2 (non-clinical) “It’s a source of pride and is a clear example of the organization investing in staff wellness.”

P25 (clinical) I think it's pretty much in line with how I already felt that the organization really does try to look at ways to support their employees and whether that be implementing something new like service dog or something like adjusting a policy or creating a new committee, or you know that there's constant that strive to support or at least have pillars in place to support employees.

## Theme 5:The Need for Organizational Improvements Beyond the Facility Dog

While many participants reported that Ember’s presence has provided them with support in the workplace, some indicated that further changes are needed to have a lasting impact. Some participants expressed that specific structural changes within the organization are necessary for overall organizational support. They felt that some policies implemented over the years required re-evaluation with staff feedback. They expressed a need for CMH to focus on improving staffing shortages to maintain good-quality patient care. Additionally, some participants reported a need for efficient communication from CMH concerning staff-reported issues.

P16 (clinical) . . .It has to be more than just the dog, and even though I think it's a great addition. I think there's lots of things that CMH does to try to promote Wellness for staff.

P21 (non-clinical) . . .I guess listening to what employees have to say and focus on coming up with resolutions to the issues that they bring up, doesn't mean positive or negative resolution, but just resolving the issues as they're identified. Communications, so communicating freely, the information that they're able to communicate in a timely manner.

## Discussion

This qualitative study explored hospital employees’ experiences with the facility dog, Ember, focusing on their perceptions of organizational support. The results revealed that Ember’s presence offered a unique type of support that is both immediate and multisensory, and led to positive organizational perceptions by fostering a sense of community and normalizing microbreaks. Although described as successful, Ember’s overall impact was deemed short-term, with some barriers hindering consistent interaction between clinical staff and Ember. The results also revealed a sense of pride among CMH staff in the organization for leading this initiative, along with an acknowledged need for more support.

The study highlights that Ember is important to participating CMH staff, as she provides immediate in-the-moment support, especially during critical debriefs, advancing the research on human-animal relationships from an organizational perspective. In the current healthcare system, healthcare workers are managing an increased workload with staffing shortages.^1,22^ Consequently, healthcare workers are working harder and longer hours, serving as a barrier to self-care, including seeking mental health support.^1,23^ While Ember’s role is not meant to replace professional mental health support, participants still reported experiencing positive benefits. This aligns with the human-animal bond theory that companion animals can serve as a social support for humans and improve their psychological wellbeing.^24^ A study involving hospital-based nurses found that canine-assisted therapy reduced anxiety by 19%.^25^ Over 20% of the nurses reported feeling workplace anxiety and nervousness before the program, which declined to 0% after interacting with a therapy dog. Another study conducted with healthcare workers in oncology units revealed a significant improvement in the participants’ mood, indicating an increase in positive feelings, such as calmness and happiness, while reducing symptoms of depression, fatigue, and tension.^26^ These healthcare workers also self-reported reduced physical symptoms of stress, such as dry mouth, headaches, and elevated heart rate. The present study’s findings, along with previous literature, highlight that facility dogs may improve the emotional and physical well-being of healthcare workers.

From an organizational perspective, Ember’s presence fostered a sense of community among CMH staff members by enhancing work engagement. This initiative was timely as Ember arrived following the peak of the COVID-19 pandemic. Pandemic-related research has highlighted that healthcare workers often worked in isolation, leading to work disengagement and limited opportunities for social connection with peers, with some remnants persisting even after the pandemic’s peak.^27,28^ Recognizing the impact of the pandemic on engagement levels of hospital staff, it is possible that Ember’s presence helped normalize and reintroduce staff engagement by motivating staff to take microbreaks. Providing employees with opportunities to pause and connect with their peers through sharing stories of their pets, personal experiences with Ember, and interacting with Ember’s handlers. This allowed participants to build and strengthen workplace relationships through increased socialization, creating a supportive work environment and boosting morale.

Our study highlights the importance of organizations promoting microbreaks, irrespective of workload. Staffing shortages hinder task management, leading to incomplete work, workflow interruptions, and compromised care standards, leaving little time for breaks.^29^ In this study, Ember’s presence helped participants take frequent, enjoyable microbreaks, often described as a good distraction, consistent with other studies with facility dogs.^9,12^ Microbreaks have been found to help reduce job stress and enhance work engagement,^30^ while adequate microbreaks enable healthcare workers to replenish energy and sustain performance.^31^ One study highlighted that longer durations of microbreaks in a healthcare setting increase staff performance by improving vigor and decreasing fatigue.^32^ While Ember’s presence facilitated opportunities for microbreaks, organizations should recognize the value of microbreaks irrespective of a facility dog’s presence. Our findings also revealed that Ember provided short-term relief, which participants described as necessary given their demanding schedules. One study highlighted that healthcare workers experiencing a heavy workload daily are 3 and a half times more likely to report emotional exhaustion than their less busy counterparts.^33^ However, these short-term benefits appeared more accessible to non-clinical staff, as clinical participants reported barriers such as heavy workloads and scheduling constraints that limited their ability to engage. The healthcare system's increased workload and staffing shortages often prevent healthcare staff from seeking time-efficient mental health support.^1,23^ highlighting the need to embed microbreaks into the work culture. This can be achieved through a facility dog such as Ember, who may provide adequate support to healthcare workers when needed, or through other organizational initiatives that allow staff to take frequent microbreaks.

The organization’s initiative to introduce Ember inspired participating CMH staff to take pride in being the first healthcare institution in Canada to introduce an NSD facility dog. It highlighted the significance of an organization’s role in maintaining a healthy workplace environment, which is not only necessary for employees to have a positive emotional and attitudinal relationship with the workplace but also to make them feel valued and supported at work. These feelings have been shown to foster a strong commitment to customer service, creativity, and employee retention in the workplace.^34^

Although our findings highlight the facility dog’s success in the hospital environment, participants emphasized that organizational improvements must be ongoing and cannot be achieved through a single initiative. They identified persistent staffing shortages and the need for better communication to ensure that employees feel heard and that their concerns are acknowledged. While these 2 common issues may appear distinct, they are interconnected. Specifically, when employees feel dismissed or ignored, their intention to leave increases^35^; conversely, feeling heard and seeing responsive action enhances perceived organizational support and strengthens commitment.^36^

## Limitations and Recommendations

Although the study’s findings show favorable outcomes, the purpose of qualitative research is not to be generalized to other healthcare settings, particularly in terms of findings highlighting a human-animal bond, as all healthcare settings may not share the same culture or policies on employing facility dogs. Furthermore, it is essential to account for self-selection bias in the data, as all participants reported mostly positive experiences with Ember. Therefore, further research employing mixed methods is required.

Through Ember’s involvement, our study revealed that organizational support may play an important role in employer-employee relationships. However, the organization must support staff beyond having a facility dog, which our findings suggest may be achieved by normalizing a culture of microbreaks in the workplace, allowing staff to prioritize their well-being without feeling guilty, and offering opportunities for increased social engagement among staff. The present study also found that an organization must work closely with staff to improve transparency and enhance communication, which can help create better organizational policies that would, in turn, enhance employees’ perceived organizational support and, by extension, commitment.^37^ In the case of introducing a facility dog, this will likely further result in equitable engagement between the facility dog and the employees, especially considering the clinical staff in the present study who faced barriers to accessing the organizational support in the form of a facility dog.

## Conclusions

The study conducted with participating CMH staff highlighted the potential impact of a facility dog, as participants expressed feeling an overall positive experience from this unique type of support. However, the study also revealed that because the impact is immediate and short-lived, and clinical staff often experience barriers to accessing the facility dog compared to their non-clinical counterparts, additional ideas and initiatives to provide further support are needed. Hence, continuing to explore integrated efforts and initiatives to improve organizational support is necessary, which may include employing a facility dog for the right healthcare environment.

## Supplemental Material

sj-docx-1-inq-10.1177_00469580261417528 – Supplemental material for A Qualitative Inquiry to Understand the Impact of a Facility Dog on Hospital Workers’ Perceived Organizational Support Supplemental material, sj-docx-1-inq-10.1177_00469580261417528 for A Qualitative Inquiry to Understand the Impact of a Facility Dog on Hospital Workers’ Perceived Organizational Support by Jaskiran Baweja, Jason B. Coe, Maziha Kamal, Janine Noorloos, Marianne Saragosa, Behdin Nowrouzi-Kia and Basem Gohar in INQUIRY: The Journal of Health Care Organization, Provision, and Financing

## References

[1] Yousefi V. Prevalence of burnout and impact of workload on physician wellness: a cross-sectional survey of hospitalists in British Columbia, Canada. J Hosp Med. 2025;20(7):688-700. doi:10.1002/jhm.13577 39741466 PMC12217418

[2] Gohar B Larivière M Lightfoot N Wenghofer E Larivière C Nowrouzi-Kia B. Meta-analysis of nursing-related organizational and psychosocial predictors of sickness absence. Occup Med. 2020;70(8):593-601. doi:10.1093/occmed/kqaa144 33313909

[3] Roth L Le Saux C Gilles I Peytremann-Bridevaux I. Factors associated with intent to leave the profession for the allied health workforce: a rapid review. Med Care Res Rev. 2024;81(1):3-18. doi:10.1177/10775587231204105 37864432 PMC10757398

[4] Nakweenda M Anthonie R van der Heever M. Staff shortages in critical care units: critical care nurses experiences. Int J Afr Nurs Sci. 2022;17:100412. doi:10.1016/j.ijans.2022.100412

[5] Chatzittofis A Constantinidou A Artemiadis A Michailidou K Karanikola MNK . The role of perceived organizational support in mental health of healthcare workers during the COVID-19 pandemic: a cross-sectional study. Front Psychiatry. 2021;12:707293. doi:10.3389/fpsyt.2021.707293 34790134 PMC8591071

[6] Pattali S Sankar JP Al Qahtani H Menon N Faizal S. Effect of leadership styles on turnover intention among staff nurses in private hospitals: the moderating effect of perceived organizational support. BMC Health Serv Res. 2024;24(1):199. doi:10.1186/s12913-024-10674-0 38355546 PMC10865721

[7] Fine AH Beck A. Understanding our kinship with animals. In: Fine AH , ed. Handbook on Animal-Assisted Therapy. Elsevier; 2010:3-15.

[8] Hosey G Melfi V. Human-animal interactions, relationships and bonds: a review and analysis of the literature. Int J Comp Psychol. 2014;27(1):117-142. doi:10.46867/ijcp.2014.27.01.01

[9] Curley T Campbell MA Doyle JN Freeze SM. First responders’ perceptions of the presence of support canines in the workplace. J Police Crim Psychol. 2022;37(4):804-812. doi:10.1007/s11896-021-09477-4

[10] Wojtas-Johnson G Dalstrom M Parizek R Castronovo L. Caring canines: tackling the problem of burnout among healthcare workers. Online J Rural Nurs Health Care. 2024;24(2):129-150.

[11] Rodriguez KE Bibbo J O’Haire ME. Perspectives on facility dogs from pediatric hospital personnel: a qualitative content analysis of patient, family, and staff outcomes. Complement Ther Clin Pract. 2022;46:101534. doi:10.1016/j.ctcp.2022.101534 35051806 PMC11141326

[bibr12-00469580261417528] Jensen CL Bibbo J Rodriguez KE O’Haire ME. The effects of facility dogs on burnout, job-related well-being, and mental health in paediatric hospital professionals. J Clin Nurs. 2021;30(9-10):1429-1441. doi:10.1111/jocn.15694 33555610 PMC11166410

[bibr13-00469580261417528] Snell V Williams S Goldstein E Chidley E Burns-Nader S. Examining healthcare workers’ experiences with facility dogs in pediatrics: a descriptive study. Child Health Care. 2025:1-13. doi:10.1080/02739615.2025.2495581

[14] George M Keller B Goldstein E Grissim L Boles J . Facility dog roles, responsibilities, and experiences in pediatric healthcare. Children’s Health Care. 2026;55(1):26-40. doi:10.1080/02739615.2024.2317331

[15] Assistance Dogs International. ADI Terms & Definitions. n.d. Accessed July 23, 2025. https://assistancedogsinternational.org/resources/adi-terms-definitions/

[16] Cambridge Memorial Hospital. Canada First - NSD certified Facility Dog dedicated to a Canadian hospital. 2022. Accessed July 24, 2025. https://www.cmh.org/node/218.

[17] Tong A Sainsbury P Craig J. Consolidated criteria for reporting qualitative research (COREQ): a 32-item checklist for interviews and focus groups. Int J Qual Health Care. 2007;19(6):349-357. doi:10.1093/intqhc/mzm042 17872937

[18] Patton M. Qualitative Research & Evaluation Methods: Integrating Theory and Practice. 4th ed. Sage Publications Inc; 2015.

[19] Braun V Clarke V. Using thematic analysis in psychology. Qual Res Psychol. 2006;3(2):77-101. doi:10.1191/1478088706qp063oa

[20] Braun V Clarke V. Conceptual and design thinking for thematic analysis. Qual Psychol. 2022;9(1):3-26. doi:10.1037/qup0000196

[21] *Dedoose, Cloud Application for Managing, Analyzing, and Presenting Qualitative and Mixed Method Research Data* .SocioCultural Research Consultants, LLC (SCRC); 2024. www.dedoose.com

[22] Tomblin Murphy G Birch S MacKenzie A Alder R Lethbridge L Little L . Eliminating the shortage of registered nurses in Canada: an exercise in applied needs-based planning. Health Policy. 2012;105(2-3):192-202. doi:10.1016/j.healthpol.2011.11.009 22176731

[23] White A Shiralkar P Hassan T Galbraith N Callaghan R. Barriers to mental healthcare for psychiatrists. Psychiatr Bull R Coll Psychiatr. 2006;30(10):382-384. doi:10.1192/pb.30.10.382

[24] Fine AH Mackintosh TK. Animal-assisted interventions: entering a crossroads of explaining an instinctive bond under the scrutiny of scientific inquiry. In: Friedman HS , ed. Encyclopedia of Mental Health. Elsevier; 2016:68-73.

[25] Jean-Baptiste C Massaro D Pinzon-Gonzales J , et al. Canine pet therapy to decrease nurse anxiety. Pr Implement Nurs Sci. 2024;3(2):19-20. doi:10.29024/pins.75

[26] Craig J Kaplan C Rode D Stojanowski M Smith C Cohen B. The impact of facility dog programming on mood and stress among healthcare workers in adult inpatient oncology units. Pr Implement Nurs Sci. 2024;3(1):16-26. doi:10.29024/pins.62

[27] Frangieh J Hughes V Edwards-Capello A Humphrey KG Lammey C Lucas L. Fostering belonging and social connectedness in nursing: evidence-based strategies: a discussion paper for nurse students, faculty, leaders, and clinical nurses. Nurs Outlook. 2024;72(4):102174. doi:10.1016/j.outlook.2024.102174 38761699

[28] Frangieh J Jones T Kinser PA Wood R Baker K. “You can’t pour from an empty cup”: a qualitative exploration of formal and informal social support as experienced by frontline nurse leaders. Nurse Lead. 2023;21(5):590-596. doi:10.1016/j.mnl.2023.07.002

[29] Wendsche J Ghadiri A Bengsch A Wegge J. Antecedents and outcomes of nurses’ rest break organization: a scoping review. Int J Nurs Stud. 2017;75:65-80. doi:10.1016/j.ijnurstu.2017.07.005 28750245

[30] Wang H Xu G Liang C Li Z. Coping with job stress for hospital nurses during the COVID-19 crisis: the joint roles of micro-breaks and psychological detachment. J Nurs Manag. 2022;30(7):2116-2125. doi:10.1111/jonm.13431 34327761

[31] Trougakos JP Hideg I Cheng BH Beal DJ. Lunch breaks unpacked: the role of autonomy as a moderator of recovery during lunch. Acad Manag J. 2014;57(2):405-421. doi:10.5465/amj.2011.1072

[32] Albulescu P Macsinga I Rusu A Sulea C Bodnaru A Tulbure BT. “Give me a break!” A systematic review and meta-analysis on the efficacy of micro-breaks for increasing well-being and performance. PLoS One. 2022;17(8):e0272460. doi:10.1371/journal.pone.0272460 PMC9432722 36044424

[33] MacPhee M Dahinten V Havaei F. The impact of heavy perceived nurse workloads on patient and nurse outcomes. Adm Sci. 2017;7(1):7. doi:10.3390/admsci7010007

[34] Gouthier MHJ Rhein M. Organizational pride and its positive effects on employee behavior. J Serv Manag. 2011;22(5):633-649. doi:10.1108/09564231111174988

[35] Doleman G Nosaka K De Leo A. Senior registered nurses’ organisational communication satisfaction, job satisfaction, burnout, and intention to stay: a cross-sectional study of two healthcare groups. J Adv Nurs. 2025;81(5):2687-2700. doi:10.1111/jan.16687 39716439

[36] Duru DC Hammoud MS. Identifying effective retention strategies for front-line nurses. Nurs Manag. 2022;29(1):17-24. doi:10.7748/nm.2021.e1971 34490763

[37] Kamal Bahrain NN Raihan Sakrani SN Maidin A. Communication barriers in work environment: understanding impact and challenges. Int J Acad Res Bus Soc Sci. 2023;13(11):1489-1503. doi:10.6007/IJARBSS/v13-i11/19498

